# Bibliometric Indices As Indicators of Research Output: Analyzing Anesthesiologists as a Paradigm for Surgical Disciplines

**DOI:** 10.7759/cureus.53028

**Published:** 2024-01-26

**Authors:** Shooka Esmaeeli, Dhanesh D Binda, Luis F Rendon, Connor M Logan, Jacob L Leung, Hannah M Nguyen, Cara E Michael, Maxwell B Baker, Lan Xu, Ala Nozari

**Affiliations:** 1 Anesthesiology, Boston University Chobanian & Avedisian School of Medicine, Boston, USA

**Keywords:** academic productivity, bibliometrics analysis, h-index, m-quotient, research evaluation

## Abstract

Anesthesiology is one of the increasingly competitive surgical specialties with a growing emphasis on scholarly activity. A metric of productivity and citation influence, the Hirsch index (h-index), can help identify mentors capable of guiding postgraduate trainees toward successful academic achievements. This study sought to determine associations between h-indices or m-quotients and manuscript publication in anesthesiology. Using the American Society of Anesthesiologists (ASA) website, accepted abstracts from the ASA Annual Meetings from 2019 to 2021 were screened (*n*=2146). The first author (FAHi) and senior author (SAHi) h-indices, as well as the first author (FAMq) and senior author (SAMq) m-quotients, were collected for each abstract using the Scopus database. Whether an accepted abstract was subsequently published as a manuscript in a peer-reviewed journal was also noted, along with the number of days between ASA presentation and publication date. Linear and logistic regression models were used for statistical analyses. In total, 348 (34.4%) of the 1012 eligible abstracts were published as manuscripts. Mean FAHi, SAHi, FAMq, and SAMq, were significantly higher for accepted ASA abstracts that were later published in peer-reviewed journals compared to accepted abstracts that were not published (p<0.001). FAHi, SAHi, FAMq, and SAMq had significant positive associations with odds of publication (*p*=0.002; *p*<0.001; *p*=0.006; *p*<0.001, respectively). There was no statistical significance between FAHi, SAHi, FAMq, or SAMq and the number of days between ASA presentation and publication. Our study uniquely demonstrates the positive, direct association between h-indices and m-quotients with the probability of publication in anesthesiology. We propose that bibliometric indices are adapted to provide a refined perspective of a physician-scientist's capabilities. Postgraduate trainees can use these indices to discern research mentors primed to foster academic excellence.

## Introduction and background

Anesthesiology is a rapidly evolving field with an increasing emphasis on innovation and research to develop new techniques, drugs, and equipment and improve patient safety and outcomes [1]. As the competitiveness for anesthesiology residency increases, postgraduate trainees and applicants are getting increasingly involved in academic projects [2]. The most recent National Resident Matching Program data report from 2022 shows that applicants who successfully matched into anesthesiology residencies in the United States have more publications than those who went unmatched [3]. Mentorship has thus become critical in facilitating the academic careers of medical students, residents, and fellows [4]. Academic productivity is important for trainees and anesthesiology residency applicants and is increasingly used to distinguish faculty, recognize their clinical excellence, and advance their administrative and academic promotion. Moreover, it has become a critical metric utilized by universities in the evaluation and promotion of their medical staff.

Despite the persistent interest in enhancing recruitment and promotion criteria for physician-researchers in anesthesiology, challenges in assessing academic scholarship quality and capability remain [5,6]. Scholarly activity comprises publishing in peer-reviewed journals, acquiring grant funding, and mentoring students, residents, and fellows. Bibliometric analyses have been proposed as statistical citation indices to measure research output. Prior studies have recommended using the Hirsch index (h-index), defined as the number of publications h with at least h citations each, as an objective measure of research productivity and influence within a field [7]. The h-index provides some insight into an author's level of academic output, with previous studies finding positive associations between h-index and total citations, funding, and professorships in anesthesiology [8-10]. While the h-index is a well-established metric of research scholarship, important limitations must be considered. For example, citations may take several years to accumulate, favoring physician-scientists with longer careers who may have published articles earlier than their counterparts [10]. To account for the length of time, an author has been academically active; the m-quotient was developed as an alternative method for ascertaining research productivity. The m-quotient is defined as the h-index divided by the number of years since the author's first publication [11].

Numerous investigations have revealed the relationship between an anesthesiologist's h-index and career status. However, no studies have evaluated the predictive value of h-indices and m-quotients on the successful publication of a manuscript in anesthesiology. Presenting at the American Society of Anesthesiologists (ASA) Annual Meeting and subsequent manuscript publication are pivotal for American students' or residents' careers. These activities bolster professional visibility, validate research competence, foster networking opportunities, and strengthen academic credentials, which are all essential for career advancement in anesthesiology. While participation in major conferences has not been shown to be associated with publication probability [12], we wanted to ascertain whether bibliometric indices would affect an abstract's time to publication after it had been presented. This investigation aimed to characterize both the h-indices and m-quotients of first and senior authors of abstracts accepted by the ASA Annual Meetings between 2019 and 2021 in order to determine the associations between h-index or m-quotient and the time to and likelihood of manuscript publication.

This article was previously posted to the Research Square preprint server on September 11, 2023.

## Review

Materials & methods

Data Extraction

**Figure 1:**
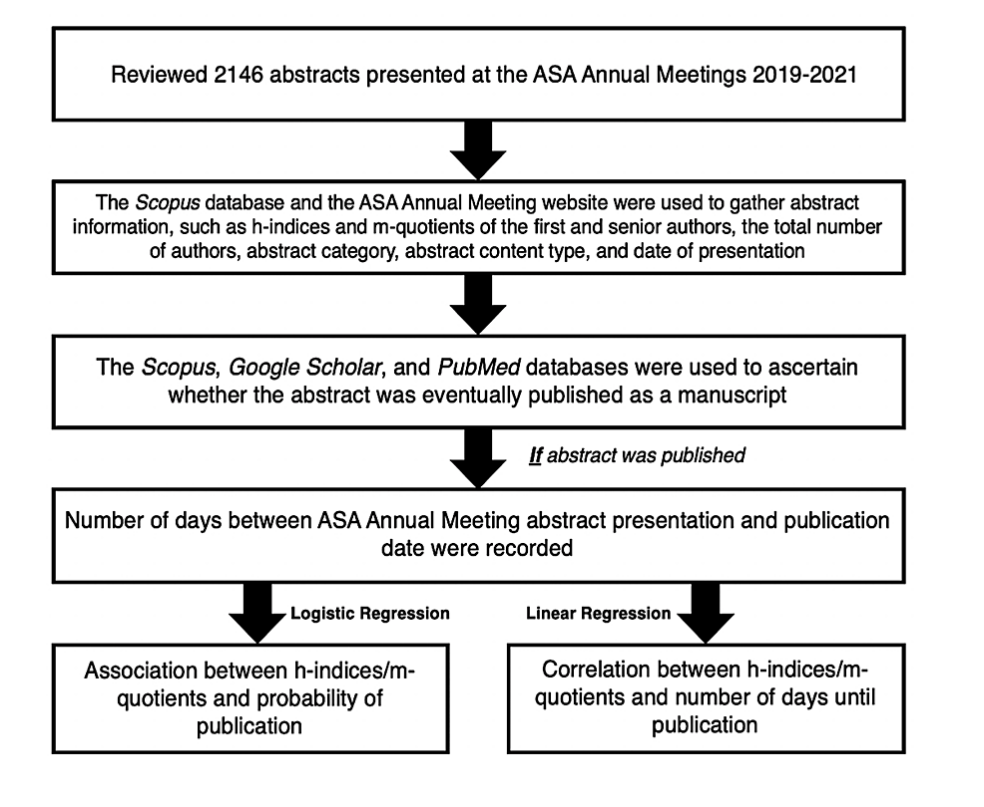
Identification and evaluation of ASA abstracts. Method of selection and evaluation of published abstracts accepted by the ASA Annual Meetings between 2019 and 2021.

For this observational bibliometric study, abstracts presented at the ASA's Annual Meetings from 2019 to 2021 were screened (n=2146). Figure 1 summarizes the identification and evaluation of the abstracts. The Scopus database collected first author h-indices (FAHi) and senior author h-indices (SAHi) for each abstract. Scopus was chosen to avoid inflated h-indices that account for unpublished materials. Given the aforementioned limitations with the h-index, we additionally chose to evaluate the m-quotient. The m-quotients of first authors (FAMq) and senior authors (SAMq) for each abstract were determined based on their h-indices divided by the years between their first publication and the abstract presentation at the ASA Annual Meeting. Abstracts where FAHi, SAHi, FAMq, or SAMq could not be determined were excluded from the analysis.

Additionally, abstracts that were published before their ASA presentation were omitted. Information about the total number of authors on each abstract, abstract content type (basic science or clinical), and year of ASA presentation was collected. Moreover, each abstract was categorized into specific topics, such as Ambulatory Anesthesia, Anesthetic Action and Biochemistry, Chronic and Cancer Pain, etc., based on the categories selected by the authors upon submission. The data also included whether the abstract was subsequently published as an original peer-reviewed journal article. If an abstract's manuscript was published based on Scopus, Google Scholar, and PubMed databases, the date of publication and the days between ASA presentation and publication were noted. Since some titles and authorship orders changed during the time between abstract presentation and journal publication, the three previously mentioned databases were cross-referenced, and the content of the published articles was reviewed to ensure the correct identification of the abstract's published manuscript. The FAHi, SAHi, FAMq, and SAMq were compiled, adhering to the authorship order in the original ASA abstract submissions.

Statistical Analysis

The authors conducted the data search and abstract screening between June 2022 and September 2022. Categorical variables are presented as counts with percentages. Continuous variables are presented as means with standard deviations (SD) and medians with interquartile ranges (IQRs). Statistical significance was defined as p<0.05. Logistic regression modeling was used to estimate the odds of abstract publication given FAHi, SAHi, FAMq, and SAMq. Linear regression modeling was used to estimate the associations between FAHi, SAHi, FAMq, and SAMq and the days between the ASA presentation and publication date. Two logistic regression models were applied since h-indices and m-quotients were highly correlated. Statistical analyses were performed using R software [13].

Results

Abstract Characteristics

**Figure 2:**
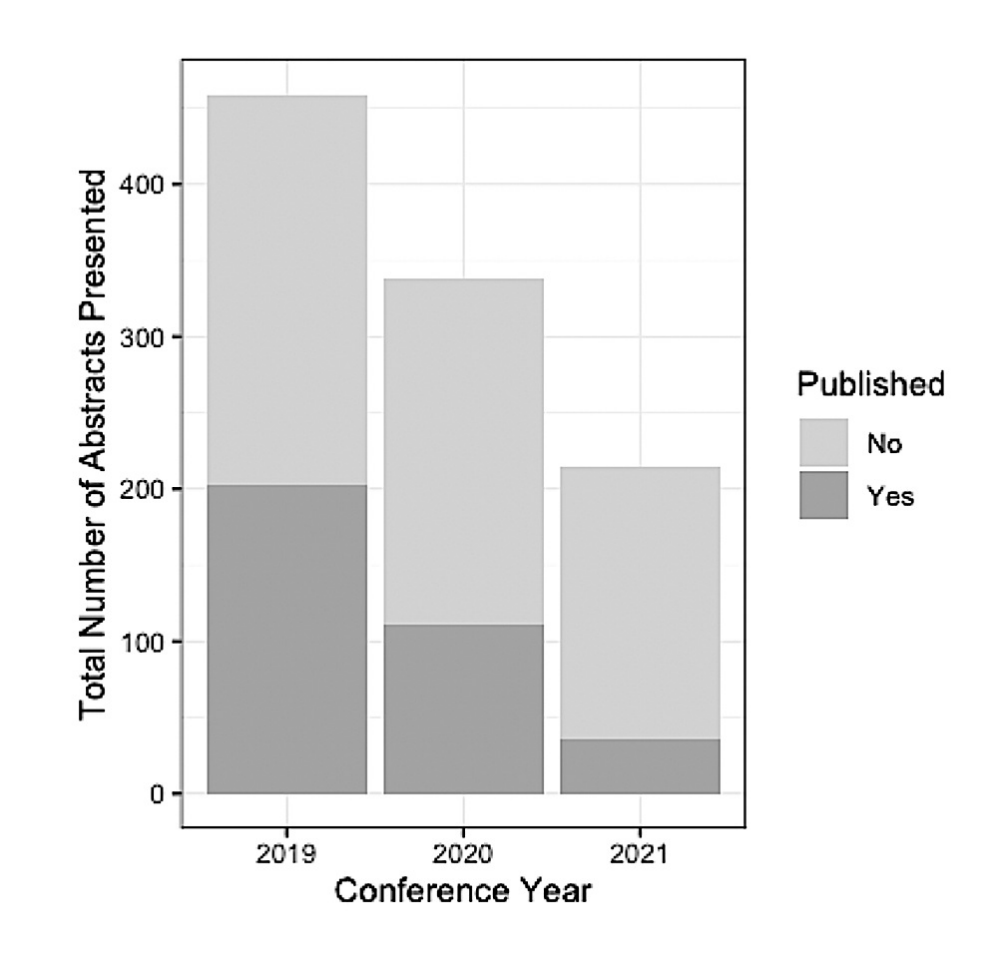
Presented ASA abstracts between 2019 and 2021. Bar graph of the total number of abstracts presented each year between 2019 and 2021 with the proportion of unpublished and published abstracts represented within each bar.

**Figure 3:**
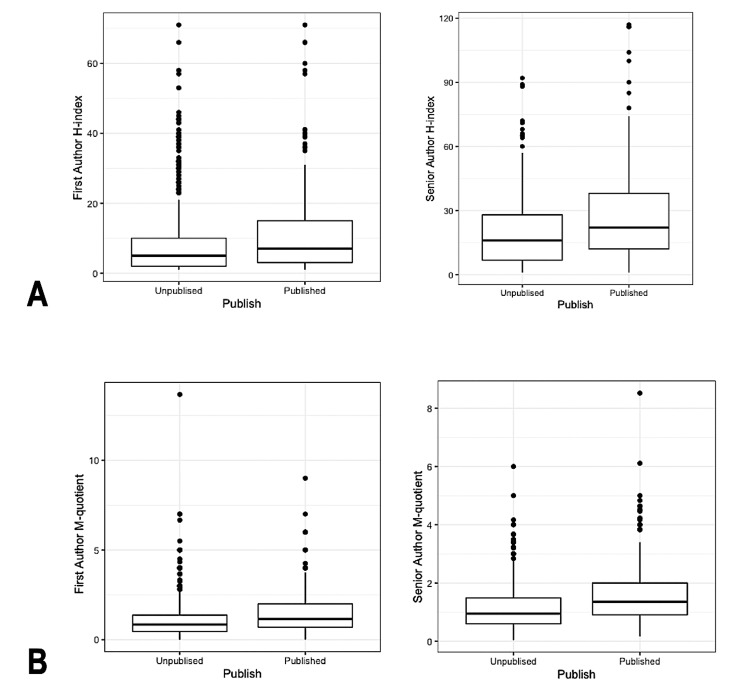
Box-and-whisker plots for senior author h-indices and m-quotients. Figure 3A. Box-and-whisker plots of the published versus unpublished abstracts based on First Author (left) and Senior Author (right) h-indices. Figure 3B. Box-and-whisker plots of the published versus unpublished abstracts based on First Author (left) and Senior Author (right) m-quotients.

A total of 2,146 abstracts were accepted by the ASA Annual Meetings from 2019 to 2021. After excluding all missing and negative values in h-index and m-quotient scores, there were 1012 abstracts, of which 348 (34.4%) were published as manuscripts (Figure 2). The median and IQR of FAHi and SAHi for published abstracts were 7.00 (IQR 3.00-15.00) and 22.00 (IQR 12.00-38.00), respectively (Figure 3A). The median and IQR of FAHi and SAHi for unpublished abstracts were 5.00 (IQR 2.00-10.00) and 16.00 (IQR 6.75-28.00), respectively (Figure 3A). The median and IQR of FAMq and SAMq for published abstracts were 1.16 (IQR 0.70-2.00) and 1.36 (IQR 0.91-2.00), respectively (Figure 3B). The median and IQR of FAMq and SAMq for unpublished abstracts were 0.85 (IQR 0.46-1.38) and 0.95 (IQR 0.60-1.49), respectively (Figure 3B). Eight hundred and thirty-nine (82.9%) abstracts were accepted as clinical research, and 173 (17.1%) were accepted as basic science research. The number of abstracts for each category is summarized in Table 1. Respiration was the category with the highest publication rate (54.8%), whereas Chronic and Cancer Pain had the lowest (11.1%). There was a significant difference in publication rate between abstract categories (X2(18)=47.7, p<0.001). Mean FAHi, SAHi, FAMq, and SAMq, were significantly higher for accepted ASA abstracts that were subsequently published in peer-reviewed journals compared to accepted abstracts that were not published (p<0.001, Table 2). The average number of days between ASA abstract presentation and publication in a peer-reviewed journal was 367.3±250.1 days.

**Table 1:** ASA abstracts from 2019 to 2021. Descriptive statistics on the abstracts gathered from ASA Annual Meetings from 2019 to 2021.

Variable	Publication		
	Yes	No	Overall
Research Type, No. (%)			
Basic Science	67 (38.7%)	106 (61.2%)	173 (17.1%)
Clinical	281 (33.5%)	558 (66.5%)	839 (82.9%)
Research Topic, No. (%)			
Ambulatory Anesthesia	7 (25.9%)	20 (74.1%)	27 (2.7%)
Anesthetic Action and Biochemistry	8 (34.8%)	15 (65.2%)	23 (2.3%)
Chronic and Cancer Pain	5 (11.1%)	40 (88.9%)	45 (4.4%)
Clinical Circulation	14 (31.1%)	31 (68.9%)	45 (4.4%)
Clinical Neurosciences	5 (16.7%)	25 (83.3%)	30 (3.0%)
Critical Care	34 (41.5%)	48 (58.5%)	82 (8.1%)
Drug Disposition	10 (43.5%)	13 (56.5%)	23 (2.3%)
Equipment, Monitoring and Engineering Technology	20 (26.7%)	55 (73.3%)	75 (7.4%)
Experimental Circulation	15 (44.1%)	19 (55.9%)	34 (3.4%)
Experimental Neurosciences	21 (40.4%)	31 (59.6%)	52 (5.1%)
Geriatric Anesthesia	4 (22.2%)	14 (77.8%)	18 (1.8%)
History and Education	9 (20.0%)	36 (80.0%)	45 (4.4%)
Information Management and Database Research	23 (41.1%)	33 (58.9%)	56 (5.5%)
Obstetric Anesthesia	19 (33.3%)	38 (66.7%)	57 (5.6%)
Patient Safety, Practice Management	15 (20.8%)	57 (79.2%)	72 (7.1%)
Pediatric Anesthesia	29 (39.7%)	44 (60.3%)	73 (7.2%)
Perioperative Medicine	54 (43.2%)	71 (56.8%)	125 (12.4%)
Regional Anesthesia and Acute Pain	39 (39.4%)	60 (60.6%)	99 (9.8%)
Respiration	17 (54.8%)	14 (45.2%)	31 (3.1%)

**Table 2:** H-indices and m-quotients for ASA abstracts. Differences in mean and median FAHi, SAHi, FAMq, and SAMq between published and unpublished abstracts. Note: FAHi (First Author H-index), SAHi (Second Author H-index), FAMq (First Author M-quotient), SAMq (Second Author M-quotient), SD (Standard Deviation), IQR (Interquartile Range).

Bibliometrics, Mean (SD); Median (IQR)	Publication		
	Yes	No	p value
FAHi	11.09 (11.44); 7.00 (3.00-15.00)	8.53 (10.35); 5.00 (2.00-10.00)	<0.001
SAHi	26.86 (20.14); 22.00 (12.00-38.00)	19.24 (15.95); 16.00 (6.75-28.00)	<0.001
FAMq	1.47 (1.17); 1.16 (0.70-2.00)	1.10 (1.10); 0.85 (0.46-1.38)	<0.001
SAMq	1.57 (1.01); 1.36 (0.91-2.00)	1.11 (0.74); 0.95 (0.60-1.49)	<0.001

Data Regression

Table 3 summarizes the results of the regression analyses. FAHi, SAHi, FAMq, and SAMq were all statistically significant predictors of abstract publication in a peer-reviewed journal (p=0.002; p<0.001; p=0.006; p<0.001, respectively). For every unit increase in FAHi, the odds of publication increased by 2% (OR=1.02, p=0.002), while every unit increase in SAHi increased the odds by 3% (OR=1.03, p<0.0001). For every unit increase in FAMq, the odds of publication increased by 19% (OR=1.19, p=0.006), while every unit increase in SAMq increased the odds by 80% (OR=1.80, p<0.0001). There was no statistically significant association between FAHi, SAHi, FAMq, and SAMq with the number of days between ASA presentation and subsequent publication (p=0.070; p=0.270; p=0.270; p=0.650, respectively).

**Table 3:** Logistic and linear regression predicting abstract publication. Results of the logistic regression of FAHi, SAHi, FAMq, and SAMq predicting abstract publication, and results of the linear regression associating FAHi, SAHi, FAMq, and SAMq with the number of days until article publication. Model I included only the covariates FAHi and SAHi. Model II included only the covariates FAMq and SAMq. Note: FAHi (First Author H-index), SAHi (Second Author H-index), FAMq (First Author M-quotient), SAMq (Second Author M-quotient), OR (Odds Ratio).

Variable	Odds ratio of abstract publication		Number of days until publication	
	OR (95% CI)	p-value	Estimate	p-value
Model I				
FAHi	1.02 (1.01, 1.03)	0.0020	-2.10	0.07
SAHi	1.02 (1.02, 1.03)	<0.0001	0.70	0.27
Model II				
FAMq	1.19 (1.05, 1.36)	0.0060	-13.00	0.27
SAMq	1.80 (1.52, 2.14)	<0.0001	6.20	0.65

Discussion

This pioneering study elucidates the predictive power of h-indices and m-quotients on manuscript publication in anesthesiology. This critical factor can magnify professional stature, bolster academic recognition, and elevate opportunities for advancement within the specialty. Notably, a statistically significant association was demonstrated between FAHi, SAHi, FAMq, SAMq, and the probability of publication, but not with the publication timeline. Moreover, the m-quotient proved more predictive than the h-index for manuscript publication odds, suggesting its superiority in gauging an author's publication quality.

The h-index is often used to measure a researcher's scientific output and has been previously characterized in the literature for many medical specialties, including anesthesiology [8-10], ophthalmology [14], dermatology [15], and otolaryngology [16]. Our study is consistent with these findings and further demonstrates that the h-index can serve as a reliable predictor for the successful transition of abstracts presented by trainees at Anesthesiology meetings into published manuscripts.

The main strength of the m-quotient is that it factors in the time since an author's first publication, which the h-index fails to consider. The m-quotient accounts for non-tangible reasons why researchers with longer careers tend to publish more, such as having more experience in the field and a better understanding of the various journals and conferences that are relevant to the research being conducted. They may also have established relationships with editors and reviewers, which can benefit getting a paper accepted for publication [17]. Interestingly, we found that the m-quotient was more predictive than the h-index when evaluating the odds of subsequent publication in a peer-reviewed journal, which implies that m-quotients may be superior to h-indices in assessing the quality of an author's publications. Early-career researchers with high m-quotients produce highly cited articles in a shorter period, while seasoned researchers with equivalent m-quotients have taken longer to achieve the same citation impact. Not only does the m-quotient factor in the time to publication, but it can also effectively gauge the impact of an author's work in their field with regard to the number of citations over time [18].

Contrary to their predictive capacity for converting ASA abstracts into complete manuscripts, neither the h-index nor the m-quotient for first or senior authors was associated with the duration from ASA abstract presentation to its publication in a peer-reviewed journal. This finding may reflect the large variation in manuscript quality not inherently assessed by the h-index or m-quotient. For example, high-impact factor (IF) journals tend to publish articles faster than lower-IF journals because of their more extensive resources, such as peer reviewers and experienced editorial board members [19]. Given that our study did not incorporate measures of the journal IF, relying solely on these indices to predict publication timelines proves challenging. Other factors that may affect the manuscript's publication time must also be considered, including the journal's acceptance rate, its peer-review process, and the number of submissions it receives.

Studies have found that higher m-quotients were negatively associated with academic rank for interventional radiologists and vascular surgeons, while h-indices were positively associated with this variable [20,21]. Given the varying results in measuring and predicting academic productivity, the h-index and m-quotient, when combined, may illustrate a more accurate metric for scholarly work. While the m-quotient seemed to be more predictive in assessing publication potential compared to the h-index, we propose, similar to Khan et al., 2013, that a bibliometric profile incorporating multiple indices be used to measure clinician-scientist academic productivity, instead of relying on a single bibliometric index [22]. Such a profile could include factors such as an author's citation count, journal impact factor, e-index, g-index, hc-index, and others not evaluated in our study [23]. This profile could convey both the quantity and quality of publications [24,25] and be useful in determining faculty promotion [22,26].

Quantitative metrics of academic output offer tangible advantages to medical students, residents, and fellows pursuing research. These indices can help identify mentors adept at guiding trainees from the inception to the fruition of academic endeavors [27]. For researchers, not only is mentorship of trainees considered an important aspect of scholarly activity, it can also help shape the future of the medical specialties by imparting knowledge and expertise to the next generation of physician-scientists. Additionally, bibliometric indices may benefit residency program directors in assessing the research productivity of their applicants [28]. With the United States Medical Licensing Examination (USMLE) Step 1 examination's recent shift from a numeric score to a "pass or fail" system, the h-index and m-quotient emerge as potentially robust, objective tools for discerning the academic prowess of residency applicants. Fellowship programs may also choose to evaluate applicants and their research productivity based on such bibliometric profiles [29].

Interestingly, the publication rates between the Research Topics of accepted abstracts at the ASA Annual Meetings were significantly variable. For instance, abstracts submitted under Respiration and Perioperative Medicine had higher publication rates than those submitted under Clinical Neurosciences and Chronic and Cancer Pain. Although the study period coincided with the COVID-19 pandemic, it is important to note that the increased submission of abstracts in the 'Respiration' category was not primarily driven by COVID-19-related topics. Instead, the abstracts classified under "Respiration" at the ASA conferences from 2019 to 2021 predominantly focused on areas such as airway management, ventilation strategies, gas flow studies, and pulmonary complications. This indicates a broader scope of respiratory research beyond the specific context of the pandemic. Regarding research disciplines and publication rates, some areas seem to garner more published manuscripts and citations than others. Nature's papers in immunology, cancer and molecular, and cell biology published in 2003 have received a substantial number of citations, ranging between 50 and 200. However, physics, paleontology, and climatology papers have generally received less than 50 citations. It is important to consider that this phenomenon reflects the differences in the dynamics of various disciplines, including their relative sizes to one another, not the quality of their research [24]. About five times as many clinical research abstracts were accepted compared to basic science abstracts. A study examining the trends in the publication of basic science and clinical research by US investigators in anesthesia journals revealed that US anesthesia research production decreased from 2001 to 2007, with declines in basic science and clinical research [30]. However, in 2010, there was a significant increase in clinical research, suggesting a modest recovery in this area. Additionally, the COVID-19 pandemic restricted access to laboratory spaces, thereby hindering researchers from dedicating sufficient time to completing basic science projects [31,32].

The study period of 2019-2021 also included the COVID-19 pandemic, which affected how conferences and journals operated in various ways. For instance, the 2020 ASA conference was held virtually. While most virtual conference attendees have preferred in-person networking, the pandemic's constraints led to a widespread shift towards virtual formats [33]. As in-person events resume, many conferences are incorporating hybrid models, recognizing the advantages of virtual participation in terms of greater accessibility and reduced environmental impact. There was also a significant increase in the number of manuscript submissions to journals, even after excluding COVID-19-related articles [32]. This rise in submissions has been attributed to the shift in focus from laboratory or clinical work to manuscript preparation and publication due to limited access to workspaces and stay-at-home orders.

Additionally, many journals modified their editorial procedures and policies to ensure rapid dissemination of relevant information, especially for COVID-19-related articles, decreasing turnaround times by 49% on average [34]. These practices included curtailing requests for additional experiments, suspending deadlines for submitting revisions and establishing "fast lanes" for coronavirus-related research. However, this decreased submission to publication timeline was most likely influenced by the expedited handling of COVID-19-related articles, in contrast to longer processing times for manuscripts on other subjects [35]. The pandemic further underscored existing issues in the academic publishing system, highlighting aspects like the pressure to publish quickly, the pursuit of attention and citations by top medical journals, and the reinforcement of journal articles as the primary metric of academic achievement [36].

We acknowledge a few limitations to this study. The abstracts were sourced from three years of one conference, which may impact the overall generalizability of our results. However, the large number of abstracts (n=2146) analyzed covered a wide range of topics in both basic and clinical sciences from the largest anesthesiology conference in the US. Additionally, this study did not differentiate between award-winning abstracts presented at the ASA meetings and others. A comparative analysis focusing on the h-index and m-quotient scores of the authors, along with the likelihood of publication and eventual impact of these award-winning abstracts, could provide further insights into the nuances of academic recognition and its correlation with bibliometric indices. Another potential limitation is the short period between the submission of 2021 abstracts and the time when we extracted our data. It is possible that this window did not allow for the publication of manuscripts in the process of completion or submission.

Similarly, the bibliometric indices for the first and senior authors could have changed with new publications, more citations, and the passage of time. As seen in Fig 2, abstracts presented at the ASA conferences dropped by approximately 50% from 2019 to 2021. This trend most likely resulted from the COVID-19 Pandemic and may have influenced the quality of manuscripts submitted for publication [37]. Furthermore, indices of scholarly productivity, including the h-index and m-quotient, have their own inherent limitations, such as the risk of inaccuracy and inconsistent validity and applicability. This study used the Scopus database for the calculation of h-indices and m-quotients. It is possible that journal articles, primarily those included in the Google Scholar or PubMed databases, were not accounted for in Scopus author searches. A recent study found wide variations in the h-index when reviewing author profiles on Google Scholar, Web of Science, Scopus, and ResearchGate [38]. Future studies are needed to evaluate scholarly output based on different metrics and to construct more complete bibliometric profiles for researchers [22].

## Conclusions

In addition to quantifying the scholarly accomplishments of clinician-scientists, the h-index and m-quotient serve as important metrics to predict the likelihood of an abstract's evolution into a manuscript. Our research is pioneering in revealing a clear, affirmative link between h-indices and publication odds in anesthesiology, as well as highlighting the predictive potency of m-quotients, even after accounting for an investigator's age and experience. These insights can potentially reshape faculty recruitment and promotion strategies and inform trainees' choices in seeking mentors for research and education.
